# Supplementary material for: Temperature dependence of quantum oscillations from non-parabolic dispersions
Source: Nat Commun. 2021 Oct 28;12:6213. doi: 10.1038/s41467-021-26450-1 (PMC8553939; doi:10.1038/s41467-021-26450-1)
Supplement: Supplementary file 1 — Supplementary Information [file 41467_2021_26450_MOESM1_ESM.pdf]

Supplemental Material for "Temperature dependence of quantum oscillations from non-parabolic dispersions"

Chunyu Guo*†,[1] A. Alexandradinata*†,[2,3] Carsten Putzke,[1] Amelia Estry,[1] Teng Tu,[4] Nitesh Kumar,[5] Feng-Ren Fan,[5] Shengnan Zhang,[6,7] Quansheng Wu,[6,7] Oleg V. Yazyev,[6,7] Kent R. Shirer,[5] Maja D. Bachmann,[5,8] Hailin Peng,[4] Eric D. Bauer,[9] Filip Ronning,[9] Yan Sun,[5] Chandra Shekhar,[5] Claudia Felser,[5] and Philip J. W. Moll†[1]

[1]*Laboratory of Quantum Materials (QMAT), Institute of Materials (IMX), École Polytechnique Fédérale de Lausanne (EPFL), CH-1015 Lausanne, Switzerland*

[2]*Institute for Condensed Matter Theory, University of Illinois at Urbana-Champaign, Urbana, Illinois 61801, USA*

[3]*Department of Physics, University of Illinois at Urbana-Champaign, Urbana, Illinois 61801, USA*

[4]*Center for Nanochemistry, Beijing National Laboratory for Molecular Sciences (BNLMS), College of Chemistry and Molecular Engineering, Peking University, Beijing 100871, China.*

[5]*Max Planck Institute for Chemical Physics of Solids, 01187 Dresden, Germany*

[6]*Chair of Computational Condensed Matter Physics (C3MP), Institute of Physics (IPHYS), École Polytechnique Fédérale de Lausanne (EPFL), CH-1015 Lausanne, Switzerland*

[7]*National Centre for Computational Design and Discovery of Novel Materials MARVEL, École Polytechnique Fédérale de Lausanne (EPFL), CH-1015 Lausanne, Switzerland*

[8]*School of Physics and Astronomy, University of St Andrews, St Andrews KY16 9SS, UK*

[9]*Los Alamos National Laboratory, Los Alamos, New Mexico 87545, USA*

(Dated: September 23, 2021)

CONTENTS

Supplementary Note 1: Review of quantization rule and standard Lifshitz-Kosevich theory 3

A. Review of Onsager-Lifshitz-Roth quantization rule 3

B. Lifshitz-Kosevich formula for de Haas-van Alphen oscillations 3

C. Lifshitz-Kosevich formula for Shubnikov-de Haas oscillations 4

Supplementary Note 2: Extended Lifshitz-Kosevich theory for high-temperature quantum oscillations 6

A. Frequency correction for high-temperature quantum oscillations 6

B. Consistency relations for topo-fermiology 7

C. Applicability to heavy-fermion materials 9

D. Distinguishing between 3D Dirac points and nodal lines 9

Supplementary Note 3: Analysis of temperature-dependent oscillation frequency for the experimental case studies 11

A. Lifshitz-Kosevich analysis of the longitudinal magnetoresistance oscillations 11

B. Fitting of experimental results 12

C. Comparison to Landau-fan method 13

Supplementary Note 4: Distinguishing between 3D Dirac points and nodal lines in $LaRhIn_5$ 13

A. Spin-orbit energy scale and Dirac-type Fermi surfaces from band-structure calculations 13

B. Possible $\boldsymbol{k} \cdot \boldsymbol{p}$ model for 3D Dirac fermions 15

Supplementary Note 5: Further characterization of $LaRhIn_5$ 18

A. Angular dependence of Shubnikov-de Haas oscillation frequencies 18

B. Dingle analysis 18

References 19

SUPPLEMENTARY NOTE 1: REVIEW OF QUANTIZATION RULE AND STANDARD LIFSHITZ-KOSEVICH THEORY

A. Review of Onsager-Lifshitz-Roth quantization rule

The phase that an electron accumulates as it makes a cyclotron orbit around the Fermi surface must be an integer multiple of 2π, according to the Onsager-Lifshitz-Roth quantization rule[1–5]:

$$l_B^2 S + \pi + \lambda_v^s = 2\pi n, \quad n \in \mathbb{Z}, \tag{1}$$

with $l_B = (h/e|B|)^{1/2}$ the magnetic length and S describes a cross-sectional area of the Fermi pocket. The subdominant term includes a quantum Maslov correction (π for orbits deformable to a circle[5]), and a *quantization offset* λ_v^s that encodes the quantum geometry of wave functions on the Fermi surface (the Berry phase), and also the generalized Zeeman interaction of the intrinsic spin and orbital magnetic moment[3,6–8].

We have introduced an index $s = \uparrow, \downarrow$ which distinguishes orbits which are spin-degenerate; s corresponds to intrinsic spin in the absence of spin-orbit coupling, but otherwise should be interpreted as a pseudospin. For spin-split Fermi surfaces, any individual orbit with a given cross-sectional area is non-degenerate, hence the index s is unnecessary.

The valley index $v = 1..N_{val}$ distinguishes between symmetry-related orbits that are separated in k-space. In this work, we focus on time-reversal-symmetric, 3D metals which may have a crystalline center of inversion; both time-reversal and spatial-inversion symmetries invert $\boldsymbol{k} \to -\boldsymbol{k}$, thus if an orbit is not centered around an time-reversal-invariant momentum (TRIM), then it necessarily has a time-reversed-partner orbit in a different valley that encloses the same area S, as illustrated in Fig. 1 of the main text. The same figure also summarizes the total number of orbits (counting both spin and valley degrees of freedom) in each symmetry class of orbits that we consider.

B. Lifshitz-Kosevich formula for de Haas-van Alphen oscillations

For 3D metals, the Fermi energy may be considered fixed up to small corrections $\propto B^{3/2}$[9]. The Onsager-Lifshitz-Roth quantization rule [cf. Supplementary Eq. (1)] gives the field B_n

at which the n'th Landau level crosses a fixed Fermi level; such crossings occur periodically in $1/B$ with frequency $F_0(E_F) = \hbar S(E_F)/2\pi e$, leading to oscillations in thermodynamic state variables such as magnetization, known as the de Haas-van Alphen effect[10]. For 3D non-magnetic metals, the oscillatory component of magnetization has the form[3,4]:

$$\Delta M \propto \sqrt{B} \sum_{r=1}^{\infty} \cos(r\lambda) \, \frac{R_D^r R_T^r}{r^{3/2}} \sin\left[r\left(2\pi \frac{F_0}{B} + \pi\right) + \phi_{LK} \right]. \tag{2}$$

For brevity, we have omitted a proportionality constant that is field- and temperature-independent; this constant is irrelevant for the purpose of topofermiology. Above, $\phi_{LK} = +\pi/4$ $(-\pi/4)$ for a minimal and maximal orbit respectively[11]. While Supplementary Eq. (2) sums oscillations from all symmetry-related orbits (counting spin and valley degrees of freedom) with possibly distinct offsets λ_s^v, the symmetry analysis of Ref. 4 shows that the time reversal symmetry imposes a zero-sum rule: $\sum_{s,v} \lambda_s^v = 0 \text{ mod } 2\pi$, and moreover there is only one independent λ_s^v which we will denote by λ; λ enters Supplementary Eq. (2) as a cosine pre-factor. The presence of higher harmonics ($r > 1$) results from the Landau-level density of states being sharply peaked. This sharpness is partially smeared due to the finite quasiparticle lifetime τ and nonzero temperature T, which result in the well known Dingle amplitude factor and thermal damping factor respectively:

$$R_D^r := \exp\left(-\frac{r}{2}\frac{h}{\varepsilon_c \tau}\right), \quad R_T^r := \frac{a_r}{\sinh(a_r)}, \quad a_r := \frac{2\pi^2 r k_B T}{\varepsilon_c}, \tag{3}$$

with $\varepsilon_c = \hbar|eB|/m_c$ the cyclotron energy, $m_c = (2\pi)^{-1}\partial S/\partial E$ the cyclotron mass, and τ the quantum lifetime.

C. Lifshitz-Kosevich formula for Shubnikov-de Haas oscillations

Compared to thermodynamic oscillations, quantum oscillations in transport coefficients, known as the Shubnikov-de Haas effect[12], are typically harder to interpret owing to multiple scattering mechanisms. We focus only on the subset of transport coefficients whose quantum oscillations plausibly have the Lifshitz-Kosevich form, so that standard routines exist for extracting Fermi-surface cross sections and cyclotron masses in the Lifshitz-Kosevich phenomenology.

In this regard, the longitudinal magnetoconductance is more amenable to Lifshitz-Kosevich analysis than the transverse magnetoconductance. Oscillations of the latter

coefficient are known to have non-Lifshitz-Kosevich corrections due to intra-Landau-level scattering – this becomes significant for lower Landau levels and for higher harmonics[13].

Though the longitudinal magnetoconductance is not directly measured, its value may be inferred from a measurement of the longitudinal magnetoresistance – assuming the field-dependent resistivity tensor elements $\rho_{i\|B,j\perp B}$ are small compared to other elements; here, i lies in the field direction but j does not, and these elements are sometimes called the planar Hall elements. (This assumption certainly holds for isotropic bands[14], and may be checked empirically on a case-by-case basis, as exemplified for $LaRhIn_5$ in Sec. .) Then the longitudinal resistivity reduces to the inverse of the longitudinal conductivity: $\sigma_{\|} \approx 1/\rho_{\|}$. The reason is that the conductivity tensor becomes block-diagonal with respect to longitudinal and transverse directions, and the longitudinal block is one-by-one and trivially inverted. It follows that the ratio of quantum-oscillatory to semiclassical (non-oscillatory) components satisfy:

$$\frac{\Delta\rho_{\|}}{\rho_{\|}^{0}} = -\frac{\Delta\sigma_{\|}}{\sigma_{\|}^{0}} + O\left(\left[\frac{\Delta\sigma_{\|}}{\sigma_{\|}^{0}}\right]^{2}\right). \tag{4}$$

For the longitudinal magnetoconductance, it is known for certain classes of Fermi surfaces that the effect of the magnetic field on localized-impurity scattering is given entirely through its effect on the density of states, whose oscillatory behavior is known to have a Lifshitz-Kosevich form. Consequently, $\Delta\rho_{\|}/\rho_{\|}^{0}$ would also have a Lifshitz-Kosevich form:

$$\Delta\rho_{\|}/\rho_{\|}^{0} \propto \sqrt{B}\sum_{r=1}^{\infty}\frac{R_D^r R_T^r}{r^{1/2}}\cos(r\lambda)\cos\left[r\left(2\pi\frac{F_0}{B}+\pi\right)+\phi_{LK}\right]. \tag{5}$$

This formula has been derived for parabolic bands in the effective-mass approximation, and is also valid for scattering off acoustic phonons at high temperatures (compared to the phonon energy)[13,15]. To our present knowledge, the explicit derivation of Supplementary Eq. (5) for Dirac-Weyl Fermi surfaces does not yet exist and would represent a significance advance.

SUPPLEMENTARY NOTE 2: EXTENDED LIFSHITZ-KOSEVICH THEORY FOR HIGH-TEMPERATURE QUANTUM OSCILLATIONS

A. Frequency correction for high-temperature quantum oscillations

The Lifshitz-Kosevich formulae in Supplementary Eq. (2) and Supplementary Eq. (5) are leading terms of an asymptotic expansion in powers of k_BT/E_F – the degeneracy parameter of Fermi gases. We now extend the Lifshitz-Kosevich formula to the next order in k_BT/E_F, to manifest the energy dependence of the cyclotron mass: $\partial m_c/\partial E$.

Following a natural generalization of Roth's derivation in Ref. 3, this extension amounts to replacing $R_T^r \sin[r(2\pi F_0/B + \pi) \pm \phi_{LK}]$ in Supplementary Eq. (2) by

$$\text{Imag}\left\{ e^{ir(2\pi F_0/B+\pi)+i\phi_{LK}} \sum_{p=0}^{\infty} 2a_r e^{-a_r(2p+1)-ib_r(2p+1)^2} \right\}; \quad b_r := \pi^3 r \frac{(kT)^2}{\varepsilon_c m_c}\left|\frac{\partial m_c}{\partial \varepsilon}\right|, \tag{6}$$

and a_r defined in Supplementary Eq. (3); note $b_r/a_r \sim k_BT/E_F$ for the Dirac-Weyl fermion.

While this form holds for arbitrary ratios of k_BT vs the cyclotron energy (with the constraint k_BT/E_F is small), it is typically the high-temperature regime (defined by $2\pi^2 k_BT/\varepsilon_c \gtrsim 1$) that is most amenable to experimental analysis. In this regime where the high Landau levels are smeared out by temperature, only the fundamental harmonic ($r = 1$) and the $p = 0$ term in Supplementary Eq. (6) survives, hence the sole correction to the Lifshitz-Kosevich formula is a T^2-correction to the oscillation frequency:

$$F_0(\mu) \to F(\mu, T) = F_0(\mu) - \frac{\pi^2}{4}\frac{(k_BT)^2}{\beta}\left|\frac{\partial(\log m_c)}{\partial E}\right|, \tag{7}$$

with μ the chemical potential and $\beta := e\hbar/2m_c c$ the effective Bohr magneton. The above effective-mass correction to the frequency holds generally for any Fermi surface; when applied to the Dirac-type Fermi surface, the result matches a calculation by Küppersbusch and Fritz for 2D Dirac systems[16]; however their proposed Lifshitz-Kosevich formula for 2D Dirac systems is discontinuous in T and μ, owing to a choice of integration path (vertical line in Fig. 2 of Ref. 16) that non-generically intersects poles of their integrand. In comparison, the Lifshitz-Kosevich formula that is presented here is continuous in T and μ. An analogous frequency correction was also calculated for graphene by Fortin and Audouard[17], however their proportionality factor for the T^2-correction is erroneously larger by a factor of two.

Beside the explicit T-dependence of $F(\mu, T)$ through the correction term in Supplementary Eq. (7), there is additionally an implicit T-dependence through $\mu(T)$ for systems of

fixed particle density. The leading term of a Sommerfeld expansion gives

$$\mu(T) = E_F - \frac{1}{6}(\pi k_B T)^2 \frac{g'(E_F)}{g(E_F)} + O(T^4), \tag{8}$$

where $g(\varepsilon)$ is the zero-field density of states, with g' the derivative of g with respect to energy. The effects of Supplementary Eqs. (7)-(8) are combined for a topo-fermiological analysis in Sec. .

Other possible mechanisms of temperature dependence are reviewed in Ref. 9. Electron-phonon interactions and thermal expansion of the solid (as contributed by lattice vibrations) are known to lead to a T^4-dependence[18], which is separable (in data analysis) from the T^2-dependence identified in Supplementary Eqs. (7)-(8). Barring ferromagnetic metals[19], we know of only one other mechanism which in principle leads to a T^2-dependence: namely, the entropic contribution of Fermi-liquid quasiparticles gives a linear-in-T term in the thermal expansion tensor[20], which implies a T^2 correction to F – assuming that the change in F is linearly related to the change in volume of solid (or its strain parameter). In most metals, the thermal expansion is dominantly driven by lattice, the oscillation frequency therefore shows a clear T^4 temperature dependence, and the changes in frequency are at most parts in 10^4 over the temperature range that quantum oscillations are observable[9]. There exists one known case of a significantly larger effect for the 'needle' pocket of Zinc[21] where both electronic and lattice contributions are observable, evident by the temperature dependence of oscillation frequency which contains both the T^2 and T^4 terms. This rare example demonstrates that for nearly-free-electron metals, the dimensions of small pockets near the Brillouin-zone boundary can be disproportionately sensitive to small changes in lattice dimensions[22]. Therefore when oscillation frequency no longer shows a simple T^2-dependence, one needs to be careful of attributing the frequency change to a topological origin.

B. Consistency relations for topo-fermiology

Assuming that the effect of thermal expansion is negligible, we derive consistency relations between the T^2 coefficient of F and its zero-temperature extrapolation – these relations being different for parabolic and linear bands allow to distinguish between Schrödinger- and Dirac-type pockets.

Let us first consider the case where a small Fermi pocket under study coexists with other Brillouin-zone-sized pockets. Then an order of magnitude estimate for g'/g in Supplementary Eq. (8) is given by the inverse of the bandwidth E_{bw}, which is typically large compared to both k_BT (the standard degeneracy condition) and the Fermi energy E_F of the small pocket (measured from band bottom or the Dirac-Weyl node, depending on context). This implies the Sommerfeld correction of μ is small compared to the Fermi energy: $\delta\mu/E_F \sim (k_BT)^2/(E_F E_{bw})$. The Sommerfeld correction to the frequency is of the order of $(k_BT)^2/(\beta E_{bw})$, with β the effective Bohr magneton of the small pocket; in comparison, the frequency correction to the frequency [Supplementary Eq. (7)] is larger by a factor of E_{bw}/E_F for the Dirac-type pocket with linear dispersion. For these reasons, we ignore the Sommerfeld correction to μ in our analysis of the multi-band metal $LaRhIn_5$. Substituting Eq. (1) of the main text into Supplementary Eq. (7) and replacing E_F with $|E_F| = 2e\hbar F_0/m_c$ for a Dirac pocket, we derive for the Dirac-type pocket a consistency relation between the T^2 coefficient of F and its zero-temperature extrapolation:

$$\text{Linear; coexisting large pocket:} \quad F(\mu,T) - F_0 \approx \Delta F^{top} = -\frac{1}{16}\frac{(\pi k_B T)^2}{\beta^2 F_0}. \tag{9}$$

Lastly, we consider semimetals with either a single, small Fermi pocket encircling a TRIM or a pair of time-reversal-related pockets as applicable to Cd_3As_2 and Bi_2O_2Se. Assuming there are no other Fermi surfaces, the oscillation contains only a single frequency and its higher harmonics. Then the Sommerfeld correction to frequency is comparable to the effective-mass correction, and should be accounted for in deriving a different consistency relation. In the case of the parabolic band, $g'/g = (d/2-1)/E_F$ in d spatial dimensions, leading to a nontrivial frequency correction for $d = 3$:

$$\text{Parabolic; single frequency:} \quad F(\mu,T) - F_0 = \Delta F^{s} = -\frac{1}{48}\frac{(\pi k_B T)^2}{\beta^2 F_0} + O(T^4). \tag{10}$$

In the case of linear bands, $g'/g = (d-1)/E_F$, therefore the Sommerfeld correction can be written as:

$$\text{Linear; Sommerfeld correction:} \quad \Delta F^{s} = -\frac{1}{24}\frac{(\pi k_B T)^2}{\beta^2 F_0} + O(T^4); \tag{11}$$

for $d = 3$. Summing the Sommerfeld correction with the effective-mass correction, the net frequency shift for linear bands is

$$\text{Linear; single frequency:} \quad F(\mu,T) - F_0 = -\frac{5}{48}\frac{(\pi k_B T)^2}{\beta^2 F_0} + O(T^4). \tag{12}$$

We see that the coefficient of the T^2 term is larger by a factor of five compared to the parabolic case [cf. Supplementary Eq. (10)].

C. Applicability to heavy-fermion materials

Our method is generally applicable to materials with large cyclotron mass. First of all, $\Delta F/F_0$ gives relative shift of frequency. For this shift to be observable, amplitude of oscillations must not be overly diminished by thermal damping. Hence we consider the product $-(\Delta F/F_0)(A/A_0)$. As shown in Fig. 3, it becomes significant at elevated temperatures just before quantum oscillations vanish. This demonstrates that heavy quasiparticle mass does not preclude the observation of oscillation frequency change, it simply renormalizes the temperature scale to lower values just as it does for the quantum oscillations themselves.

The physical origin for the general applicability is that both the thermal damping of the quantum oscillation amplitude, $A(T)$, and the strength of the frequency shift, $\Delta F(T)^{top}$, are set by the same physics. Key is to compare the Landau level spacing, i.e. the cyclotron energy, to the thermal broadening of the Fermi-Dirac distribution. Therefore the cyclotron mass value only sets the temperature range at which the oscillation frequency change is best resolvable (Supplementary Fig. 1). Importantly, if quantum oscillations in a heavy fermion material can be observed at sufficient amplitude, $\Delta F(T)^{top}$ can be observed as well.

D. Distinguishing between 3D Dirac points and nodal lines

Given that a pocket is of Dirac type, one may ask a more fine-grained question of the Dirac crossing: does the cyclotron orbit (a) encircle an isolated $\boldsymbol{k}$-point (3D Dirac point) of four-fold energy degeneracy, or does it (b) link with an isolated $\boldsymbol{k}$-point (nodal line) of four-fold energy degeneracy? In the former case, the energy degeneracy splits linearly in $\boldsymbol{k}$ along all directions away from the Dirac point. In case (b), the energy degeneracy only splits linearly in two directions that are locally orthogonal to the nodal line; there is therefore a 2D Dirac fermion in any small $\boldsymbol{k}$-rectangle pierced by the line.

To distinguish between these scenarios, it is worth reviewing the role of crystallographic symmetry and the spin-orbit interaction in stabilizing either type of degeneracies. In non-magnetic, centrosymmetric metals with negligible spin-orbit coupling, it is known that band

degeneracies occur along lines, and such degeneracies are generically lifted by the spin-orbit interaction. According to degenerate perturbation theory, the energy splitting occurs to the first order in the spin-orbit coupling; in contrast, spin-orbit-induced shifts of energy levels (away from the nodal line) occur to the second order.

Suppose a cyclotron orbit is found that satisfies either Supplementary Eq. (9) or Supplementary Eq. (12) depending on context, implying that the pocket is linearly dispersing in the two directions orthogonal to the field. If we further hypothesize that this orbit links with a nodal line, we must presume that the spin-orbit splitting E_{soc} of the nodal line is so weak as to not spoil this linear dispersion. A necessary consistency condition is that

$$E_{soc} \ll |E_F| = \left| \frac{\partial E}{\partial \log m_c} \right|, \tag{13}$$

with $|E_F|$ measured from the four-fold degeneracy (within the small $\boldsymbol{k}$-rectangle pierced by the line); the second equality in Supplementary Eq. (13) follows from the linearity of the Dirac dispersion, and note $\partial E/\partial \log m_c$ is directly measurable from the T^2 dependence of the oscillation frequency.

On the other hand, certain crystallographic point-group symmetries (beyond spatial inversion) stabilize four-fold degeneracies at isolated points in $\boldsymbol{k}$-space, no matter the strength of the spin-orbit interaction. For concreteness, we focus on rotational symmetry of order greater than two, which is a symmetry of the 3D Dirac semimetals Cd_3As_2[23] and Na_3Bi[24], as well as the candidate $LaRhIn_5$. Assuming such symmetry, a Dirac-type cyclotron orbit may either encircle a Dirac point or link with a nodal line (cases a,b). If the spin-orbit energy scale is found to be comparable or greater than the experimentally attained $|E_F|$, then the nodal-line scenario is ruled out according to Supplementary Eq. (13), and the Dirac-point scenario remains as the only consistent scenario.

Unfortunately we know of no direct means to estimate E_{soc} from quantum oscillations; such quantity can in principle be measured by angle-resolved photoemission spectroscopy, or estimated from first principles – it is now routine to compare band structures (of the same material) with and without spin-orbit coupling. Such a first-principles determination of E_{soc} will be used to argue for the Dirac-point scenario for $LaRhIn_5$, in Sec. below.

SUPPLEMENTARY NOTE 3: ANALYSIS OF TEMPERATURE-DEPENDENT OSCILLATION FREQUENCY FOR THE EXPERIMENTAL CASE STUDIES

A. Lifshitz-Kosevich analysis of the longitudinal magnetoresistance oscillations

Supplementary Fig. 2 shows that for $LaRhIn_5$ the planar-Hall elements (of the longitudinal resistivity) are significantly smaller than all other elements, thus justifying Supplementary Eq. (4) and the Lifshitz-Kosevich form of the longitudinal magnetoconductance,

$$\frac{\Delta\rho_{\parallel}}{\rho_{\parallel}^{0}} = \sum_{r=1}^{\infty} A'\sqrt{\frac{B}{r}} cos(r\lambda_1^{\uparrow}) \cdot \frac{\alpha r m_c T/B}{sinh(\alpha r m_c T/B)} \cdot e^{-\frac{1140 r\sqrt{F}}{l_q B}} \cdot cos[r(2\pi\frac{F}{B} - \pi) + \phi_{LK}] \tag{14}$$

Here $\alpha = 2\pi^2 c k_B/e\hbar = 14.69\ T/K$, r is the order of harmonics, F corresponds to the oscillation frequency, l_q represents the quantum mean free path of electrons in cyclotron motion, m_c stands for the cyclotron mass in unit of free electron mass, and $\phi_{LK} = \pi/4$ is the correction phase for a minimal orbit of a 3D Fermi surface. Note that for the Dingle damping term ($e^{-\frac{1140 r\sqrt{F}}{l_q B}}$) we assume the Fermi surface is isotropic since the oscillation frequency of both Cd_3As_2 (Supplementary Fig. 3) and $LaRhIn_5$ is almost angle-independent (Supplementary Fig. 4). For Bi_2O_2Se, the Fermi surface is ellipsoidal as evident by the clear change of oscillation frequency with field applied along different crystal axis (Supplementary Fig. 3). In this case, the magnetic field is applied along the ellipsoidal axis ($B \parallel c$) to avoid possible complexity due to Fermi surface anisotropy. The entire experimental dataset measured at different temperatures is then fitted globally by a standard least squares regression method using the non-linear model fitting function provided by Mathematica. In this procedure, both the cyclotron mass (m_c) and the quantum mean free path (l_q) are set to be temperature-independent while the oscillation frequency (F) is *not* restricted to the same value at different temperatures, which directly yields the best-fit $\Delta F(T)$ to all experimental data. The error bar is determined by the standard error of the fitting parameters generated by the non-linear regression fitting procedure. The value and error bar of the fitting parameters for all three materials are listed in Table (S1). Note that for Cd_3As_2 and Bi_2O_2Se the quantization offset value cannot be determined due to the absence of higher harmonic oscillations.

Materials	Cd_3As_2	$LaRhIn_5$ (Device-1)	Bi_2O_2Se
A' (10^{-3})	118.1±4.6	2.5±0.06	36±0.03
$\lambda_1^{\uparrow}$ (π)	/	0.98±0.01	/
F_0 (T)	43.75±0.004	6.94±0.001	33.28±0.003
$\frac{dF}{d(T^2)}$ (10^{-4} T/K^2)	-0.701±0.003	-8.94±0.12	-5.02±0.1
m_c (m_e)	0.037±0.003	0.067±0.007	0.18±0.008
l_q (nm)	230.7±23	129±7	130.7±20

TABLE 1. List of values and standard errors for all fitting parameters for different materials. Here parameter $\mathrm{d}F/\mathrm{d}T^2$ (A) is produced by the polynomial fitting of temperature-dependent $F(T)$ with $F(T) = F_0 + AT^2$.

B. Fitting of experimental results

The analysis procedure described in A. has been applied to all data, measured on different materials as well as different devices. At all temperatures the experimental results can are well described by the general Lifshitz-Kosevich formula for longitudinal magneto-transport[Supplementary Eq. (5)], as presented in Supplementary Fig. 5, Supplementary Fig. 6, Supplementary Fig. 7 for Cd_3As_2, Bi_2O_2Se and $LaRhIn_5$ respectively. The temperature dependence of oscillation frequencies for different materials/devices are readily included in Fig. 4. To further demonstrate the validity of the temperature dependent frequency fitting, a log-log plot of frequency change $-\Delta F$ versus temperature for all three materials is also presented (Supplementary Fig. 9). It is clear that T^2 dependence is the only possible and sufficient description of the experimental results, the physical origin of which is described in Sec. in details. For a further consistency check, we have also performed a standard Lifshitz-Kosevich fit to the temperature depedence of quantum oscillation amplitude which are obtained via FFT analysis at certain field window (Supplementary Fig. 8). The results demonstrate the consistency in cyclotron mass value obtained from both methods. These results clearly demonstrate not only the topological fingerprint, but also the consistency of such analysis.

C. Comparison to Landau-fan method

To demonstrate the advantage of our method, we have also performed the widely-used Landau-Fan plot method to both Cd_3As_2 and Bi_2O_2Se (Supplementary Fig. 10). This method clearly fails to distinguish the topological Cd_3As_2 and topologically-trivial Bi_2O_2Se as the linearly-extrapolated residual Landau index is ≈ 0.5 for both materials. Such failure is likely resulted from the large SOC of Bi_2O_2Se which contributes strongly to the oscillation phase despite its topologically trivial nature, as elaborated in previous sections.

SUPPLEMENTARY NOTE 4: DISTINGUISHING BETWEEN 3D DIRAC POINTS AND NODAL LINES IN LARHIN$_5$

Here we address if the 7 T Dirac-type orbit of $LaRhIn_5$ encircles a 3D Dirac point or links with a nodal line, a discussion of interest for the specific physics of this compound. The nodal-line scenario was proposed by Mikitik and Sharlai[25] based on a speculation that the spin-orbit interaction is weak, but we will instead argue for a Dirac point that is stabilized by the four-fold rotational symmetry of $LaRhIn_5$. Here we demonstrate that the spin-orbit-induced energy splitting of the nodal line is at least comparable in magnitude to the Fermi energy E_F measured from the nodal degeneracy; based on arguments presented in Sec. , this rules out the nodal-line scenario.

We have previously determined from our high-temperature oscillation analysis that $|E_F| = |\partial E/\partial \log m_c| \approx 24$ meV, based on the interpretation of a Dirac-type pocket. All that remains is to show that E_{soc} is at least comparable to 24 meV, and indeed our first-principles estimate for E_{soc} gives a value ranging from $20 - 100$ meV, as described in Sec. below. To further support the Dirac-point scenario, we describe a possible $\boldsymbol{k} \cdot \boldsymbol{p}$ model in Sec. .

A. Spin-orbit energy scale and Dirac-type Fermi surfaces from band-structure calculations

Band-structure calculations were performed with the goals of estimating E_{soc} and reproducing the experimentally observed Dirac-type pocket. Using the Vienna ab initio Simula-

tion Package (VASP)[26,27], we performed a density-functional calculation within the generalized gradient approximation (GGA), with projector-augmented-wave (PAW) potentials[28,29] and a $7\times\ 7\times 5$ Γ-centered k-point grid for Brillouin zone sampling. Wave functions were expanded in a plane-wave basis up to an energy cutoff of 230 eV. If our first-principles calculation is performed ignoring the spin-orbit interaction, we find a nodal-line degeneracy within the high-symmetry, mirror-invariant planes, as illustrated by red lines in Supplementary Fig. 11(b). While, with the inclusion of spin-orbit coupling (calculated by a second-variational procedure), we show with the blue lines of Supplementary Fig. 11(b) that the nodal-line degeneracy is lifted on a scale $E_{SOC} \approx (20 - 100)$ meV.

To check for the robustness of these results against choices of functionals in the calculation, a slightly different calculation was performed in VASP, with the PAW basis sets[28] combining GGA. The exchange-correlation functional of Perdew, Burke and Ernzerhof (PBE)[30] is added in this case. The cutoff energy for the plane wave expansion was set to 500 eV and a k-point mesh of $9 \times 9 \times 6$ was used in the bulk calculations. The Irvsp code[31] was used to obtain the irreducible representations of Bloch states. The obtained band structures with and without spin-orbit coupling (SOC) are shown in Supplementary Fig. 11(c) and (d). Without SOC, the calculation confirms that $LaRhIn_5$ is a nodal-line metal, e.g., one point on this nodal line is a crossing between two irreducible representations Γ_2 and Γ_4 on the Γ-M line, as shown by the touching between blue and green energy levels in Supplementary Fig. 11(c). All band crossing are gapped when SOC is included except the one along the Γ-Z direction which is protected by C_{4v} symmetry. The irreducible representations Γ_6 and Γ_7 are degenerate at a Dirac point on the Γ-Z line, as shown in Supplementary Fig. 11(d).

While both the comparison of energy scales and symmetry analysis support the Dirac-point scenario, there are deficiencies in our band-structure calculation that prevent full confidence. Foremost is that the calculated oscillation frequencies (of the Brillouin-zone-sized pockets) deviate from the measured frequencies $[F \sim (1 - 10)kT]$ by about 10%. This makes it unlikely that the calculation would reproduce fine features of order 7 T, and indeed the calculation does not produce 3D Dirac points within a 25 meV window of the Fermi level – though it does produce 3D Dirac points at ≈ 420 meV below the Fermi level, which may or may not be a red herring; this calculated Dirac point is circled in Supplementary Fig. 11(a) and (d). Since all that our argument needs is an order of magnitude estimate for E_{soc}, the argument's conclusion should not be overly sensitive to minor deficiencies of the

calculational method. It is hoped that future refinements of the band-structure model of $LaRhIn_5$ will settle this matter conclusively.

B. Possible $\boldsymbol{k} \cdot \boldsymbol{p}$ model for 3D Dirac fermions

To further support the Dirac-point scenario, we performed a $\boldsymbol{k} \cdot \boldsymbol{p}$ analysis assuming only that the four independent wave functions (at the hypothesized Dirac crossing) are predominantly Rhodium d_{z^2} and $d_{x^2-y^2}$ orbitals, with each orbital doubled owing to spin. These same wave functions span the degenerate subspace of the calculated Dirac crossing, as described in Sec. .

Postponing the technical derivation below, we first summarize the salient conclusions of our $\boldsymbol{k}\cdot\boldsymbol{p}$ analysis: The linearized $\boldsymbol{k}\cdot\boldsymbol{p}$ Hamiltonian is simply a direct sum of two Weyl Hamiltonians with opposite chirality. Chirality being a conserved quantity is an emergent property of *small*, rotation-invariant Dirac FS's, and is not generally expected for Cd_3As_2/Na_3Bi. One implication of conserved chirality is that for a field parallel to the rotation axis c, $\lambda_1^{\uparrow} = \lambda_1^{\downarrow} = \pi$, with $\uparrow / \downarrow$ viewed as a chirality index. The π phase is completely attributed to a Berry phase; the contribution by the Zeeman interaction vanishes owing to spin being locked in the plane orthogonal to the rotation axis[4]. This linearized model gives a plausible account for the measured $\lambda_1^{\uparrow} = (0.98 \pm 0.01)\pi$; the small deviation from π is plausibly attributed to higher-order corrections in the $\boldsymbol{k} \cdot \boldsymbol{p}$ Hamiltonian. By adding a Hamiltonian term that is quadratic in k_z and proportional to the identity matrix, the Fermi surface can be transformed to have a 'neck' with a minimal cross-section at $k_z = 0$ – this would account for the Lifshitz-Kosevich phase $\phi_{LK} = +\pi/4$ for $B//c//k_z$ parallel to the rotational axis. (A similar-looking 'neck' Fermi surface is illustrated in Fig. 1(b) of Ref. 25 and derived from a different $\boldsymbol{k} \cdot \boldsymbol{p}$ model.)

Our $\boldsymbol{k} \cdot \boldsymbol{p}$ model reproduces all salient features of our data for $B//c$, but does not, however, explain the data at various tilt angles of B. As illustrated in Supplementary Fig. 4(b), a quantum-oscillation frequency F between 7 to 8 T persists over a 90° tilt in the $a-c$ crystallographic plane, suggesting a nearly isotropic Fermi surface. However, the observation of $\phi_{LK} = +\pi/4$ caution against the simplest interpretation of a closed isotropic pocket as it corresponds to a minimum instead of maximum orbit. We have not been able to infer a completely explanatory model based on the present data. However it is worth remarking

that the persistence of $F \approx 7$ T over 90° is even harder to explain in the nodal-line scenario: the Fermi surface of a nodal-line semimetal is highly anisotropic and is known (in some cases[32]) to exhibit magnetic breakdown depending on the field orientation.

For a detailed $\boldsymbol{k} \cdot \boldsymbol{p}$ analysis, let us define the tensor products $\{\sigma_i \otimes \tau_j\}$ of two Pauli matrices as a basis for Hermitian operators in the four-dimensional vector space, with the correspondence

$$\tau_3 = +1 \leftrightarrow d_{z^2}, \;\; \tau_3 = -1 \leftrightarrow d_{x^2-y^2}, \;\; \sigma_3 = +1 \leftrightarrow \uparrow, \;\; \sigma_3 = -1 \leftrightarrow \downarrow, \tag{15}$$

with $\uparrow$ / $\downarrow$ referring (in this appendix) to spin up and down along the rotation-invariant z axis. The group of the wavevector at the Dirac point includes the point group C_{4v}, which is generated by a four-fold rotation $\mathfrak{c}_{4z}$ and a reflection $\mathfrak{r}_x$ that inverts $x \to -x$. In addition, the groups of all wavevectors have the symmetry $T\mathfrak{i}$, which is the composition of time reversal with spatial inversion. In the four-dimensional vector space, the matrix representations of the symmetry generators are

$$T\mathfrak{i} = i\sigma_2 K, \quad \mathfrak{c}_{4z} = e^{-i\sigma_3\pi/4}\tau_3, \quad M_x = -i\sigma_1. \tag{16}$$

The first symmetry ensures that the $\boldsymbol{k}\cdot\boldsymbol{p}$ Hamiltonian $H(\boldsymbol{k})$ can only contain five anticommuting matrices:

$$\sigma_1\tau_2, \; \sigma_2\tau_2, \; \sigma_3\tau_2, \; \tau_1, \; \tau_3, \tag{17}$$

ignoring the identity matrix. Let us keep only terms in $H(\boldsymbol{k})$ which are independent of or linear in $\boldsymbol{k}$. The four-fold symmetry ensures that

$$H'(\boldsymbol{k}) = v_{\parallel}(k_y\sigma_1 + k_x\sigma_2)\tau_2 + v'_{\parallel}(k_x\sigma_1 - k_y\sigma_2)\tau_2 + (v_\perp k_z + \varepsilon_0)\tau_3. \tag{18}$$

The reflection symmetry allows us to set $v'_{\parallel} = 0$. Redefining the zero of k_z to lie at the Dirac point, we are left with

$$H(\boldsymbol{k}) = v_{\parallel}(k_y\sigma_1 + k_x\sigma_2)\tau_2 + v_\perp k_z\tau_3. \tag{19}$$

By a unitary transformation, the Hamiltonian is expressible as a direct sum of two (3+1)D Weyl Hamiltonian with opposite chirality:

$$U = \tfrac{1}{\sqrt{2}}(I + i\sigma_3\tau_1), \quad UH(\boldsymbol{k})U^{-1} = v_{\parallel}(k_y\sigma_1 + k_x\sigma_2)\tau_2 + v_\perp k_z\sigma_3\tau_2 := H'(\boldsymbol{k}), \tag{20}$$

with the chirality operator being τ_2. The following identity is useful to derive the above expression:

$$U = \frac{1}{\sqrt{2}}(I + iA), \quad A^2 = I, \quad UBU^{-1} = \begin{cases} B, & [A,B] = 0 \\ \frac{i}{2}[A,B], & \{A,B\} = 0. \end{cases} \tag{21}$$

with $[A,B]$ the commutator and $\{A,B\}$ the anticommutator. In the new basis, the Hamiltonian has the constraint:

$$\mathfrak{r}_z H'(\boldsymbol{k}) \mathfrak{r}_z = H'(k_x, k_y, -k_z), \quad \mathfrak{r}_z = \sigma_3 \tau_3, \tag{22}$$

even though the reflection $z \to -z$ is not in the little group of the wavevector; such an 'emergent' symmetry will be spoiled by corrections to $H(\boldsymbol{k})$ which are higher order in $\boldsymbol{k}$.

For simplicity, let us consider applying a field parallel to the rotation axis. Owing to the emergent reflection symmetry of Supplementary Eq. (22), the extremal orbit lies at $k_z = 0$, where $H'(k_x, k_y, 0) = v_{\|}(k_y \sigma_1 + k_x \sigma_2)\tau_2$ simplifies to a direct sum of two (2+1)D Dirac Hamiltonians. Because chirality is conserved under parallel transport (in this linearized approximation), the Berry phase can be calculated separately for each Dirac fermion and simply equals π. The orbital magnetic moment matrix can be expressed in terms of interband matrix elements of the position operator [see Eq. (65) in Ref. 5], however such matrix elements vanish between two states with distinct chirality. We then apply that the single-band orbital moment for a (2+1)D Dirac fermion vanishes[4]. Finally, the spin operator $S_z = (\hbar/2)\sigma_z$ in the original basis is still represented as $(\hbar/2)\sigma_z$ in the new basis. Since $[S_z, \tau_y] = 0$, the Zeeman spin interaction also does not couple states with distinct chirality; since the spin of each Dirac fermion lies in the plane orthogonal to the rotational axis (and also orthogonal to the field), the Zeeman spin interaction has a vanishing effect. In combination, the quantization offset λ simply equals a Berry phase of π, for both chiralities of Weyl fermions.

SUPPLEMENTARY NOTE 5: FURTHER CHARACTERIZATION OF LARHIN$_5$

A. Angular dependence of Shubnikov-de Haas oscillation frequencies

Fast-Fourier-Transformation (FFT) is performed to analyze the Shubnikov-de Haas (SdH) oscillations of the high frequencies belonging to the large, trivial Fermi surfaces. In contrast, the Dirac pocket was analyzed by directly fitting Supplementary Eq. (14) as appropriate for such small frequencies (see SIV). The high-frequency SdH oscillations were measured at $T = 2$ K with current applied along c-axis and field rotates from c to a-axis [Supplementary Fig. 12(a)]. At each angle the results are shifted and normalized according to the largest peak, the square root of oscillation amplitudes are taken for visualizing the small details. The frequencies corresponding to the larger Fermi surfaces α_1, β_1 and β_2 are denoted by the dashed lines. The angular dependence of oscillation frequencies [Supplementary Fig. 12(b)] are obtained by identifying the positions of major peaks in the FFT spectrum. The angular dependence of these high-frequency oscillations corresponding to the large FS cross-sections [solid symbols in Supplementary Fig. 12(b)] agrees well with previous dHvA results[33] indicated by dashed lines.

B. Dingle analysis

Clearly obserable quantum oscillations observed at $T = 2$ K with current applied along c-axis and field applied along a-axis [Supplementary Fig. 13(a)] makes it possible to determine the quantum mean free path value. FFT analysis is performed within different field windows [Supplementary Fig. 13(b)] for the quantum mean free path analysis. The enhanced peak value at larger magnetic field represents the increasing ratio between Landau level spacing and broadening due to scattering, as expected for the Dingle damping effect. The quantum mean free paths of different extremal orbits are therefore calculated by fitting the field dependence of oscillation amplitudes[Supplementary Fig. 13(c)] in consideration of both thermal and Dingle damping terms. The results yield long quantum mean free paths

(300 to 900 nm) as expected for the clean $LaRhIn_5$ sample.

---

[1] Onsager, L. Interpretation of the de Haas-van Alphen effect. London Edinburgh Philos. Mag. J. Sci. **43**, 1006–1008 (1952).

[2] Lifshitz, I. M. & Kosevich, A. M. On the theory of the de Haas-van Alphen effect for particles with an arbitrary dispersion law. Dokl. Akad. Nauk SSSR **96**, 963 (1954).

[3] Roth, L. M. Semiclassical theory of magnetic energy levels and magnetic susceptibility of Bloch electrons. Phys. Rev. **145**, 434–448 (1966).

[4] Alexandradinata, A., Wang, C., Duan, W. & Glazman, L. Revealing the topology of Fermi-surface wave functions from magnetic quantum oscillations. Phys. Rev. X **8**, 011027 (2018).

[5] Alexandradinata, A. & Glazman, L. Semiclassical theory of Landau levels and magnetic breakdown in topological metals. Phys. Rev. B **97**, 144422 (2018).

[6] Roth, L. M. Theory of Bloch electrons in a magnetic field. J. Phys. Chem. Solids **23**, 433 – 446 (1962).

[7] Chang, M. & Niu, Q. Berry phase, hyperorbits, and the hofstadter spectrum: Semiclassical dynamics in magnetic Bloch bands. Phys. Rev. B **53**, 7010–7023 (1996).

[8] Gao, Y. & Niu, Q. Zero-field magnetic response functions in Landau levels. Proc. Nat. Acad. Sci. **114**, 7295–7300 (2017).

[9] Shoenberg, D. Magnetic oscillations in metals (Cambridge university press, 2009).

[10] De Haas, W. J. & van Alphen, P. M. The dependence of the susceptibility of diamagnetic metals upon the field. Proc. Netherlands Roy. Acad. Sci. **33**, 1106 (1930).

[11] Lifshitz, L. M. & Kosevich, A. M. Theory of magnetic susceptibility in metals at low temperatures. J. Exp. Theor. Phys. **2**, 636 (1956).

[12] Shubnikov, L. W. & de Haas, W. J. New phenomena in the change in resistance of bismuth crystals in a magnetic field at the temperature of liquid hydrogen (I). Proc. Netherlands Roy. Acad. Sci. **33**, 130 (1930).

[13] Roth, L. M. & Argyres, P. N. Magnetic quantum effects-Semiconductors and Semimetals (1966).

[14] Lifshitz, I. M., Azbel', M. IA. & Kaganov, M. I. The theory of galvanomagnetic effects in metals. J. Exp. Theor. Phys. **4**, 41–54 (1957).

[15] Argyres, P. N. Quantum theory of longitudinal magneto-resistance, J. Phys. Chem. Solids **4**, 19–26 (1958).

[16] Küppersbusch, C. & Fritz, L. Modifications of the lifshitz-kosevich formula in two-dimensional Dirac systems. Phys. Rev. B **96**, 205410 (2017).

[17] Fortin, J. & Audouard, A. Effect of electronic band dispersion curvature on de Haas-van Alphen oscillations. Eur. Phys. J. B **88**, 225 (2015).

[18] Lonzarich, G. G. & Cooper, N. S. Temperature dependence of the Fermi surface of gold. J. Phys. F Met. Phys. **13**, 2241 (1983).

[19] Lonzarich, G. G. & Gold, A. V. Temperature dependence of the exchange splitting in ferromagnetic metals I. information from the de haas–van alphen effect in iron. Can. J. Phys. **52**, 694–703 (1974).

[20] Barron, T. H. K., Collins, J. G. & White, G. K. Thermal expansion of solids at low temperatures. Adv. Phys. **29**, 609–730 (1980).

[21] Berlincourt, T. G. & Steele, M. C. Temperature-dependent de Haas-van Alphen parameters in zinc. Phys. Rev. **95**, 1421–1428 (1954).

[22] O'Sullivan, W. J. & Schirber, J. E. Pressure dependence of the low-frequency de haas—van alphen oscillations in zn. Phys. Rev. **151**, 484–494 (1966).

[23] Wang, Z., Weng, H., Wu, Q., Dai, X. & Fang, Z. Three-dimensional Dirac semimetal and quantum transport in Cd_3As_2. Phys. Rev. B **88**, 125427 (2013).

[24] Wang, Z., Sun, Y., Chen, X., Franchini, C., Xu, G., Weng, H., Dai, X. & Fang, Z. Dirac semimetal and topological phase transitions in A_3Bi (A = Na, K, Rb). Phys. Rev. B **85**, 195320 (2012).

[25] Mikitik, G. P. & Sharlai, Y. V. Berry phase and de haas–van alphen effect in $LaRhIn_5$. Phys. Rev. Lett. **93**, 106403 (2004).

[26] Kresse, G. & Furthmüller, J. Efficient iterative schemes for ab initio total-energy calculations using a plane-wave basis set. Phys. Rev. B **54**, 11169–11186 (1996).

[27] Kresse, G. & Hafner, J. Ab initio molecular dynamics for open-shell transition metals. Phys. Rev. B **48**, 13115–13118 (1993).

[28] Blöchl, P. E. Projector augmented-wave method. Phys. Rev. B **50**, 17953–17979 (1994).

[29] Kresse, G. & Joubert, D. From ultrasoft pseudopotentials to the projector augmented-wave method. Phys. Rev. B **59**, 1758–1775 (1999).

[30] Perdew, J. P., Burke, K. & Ernzerhof, M. Generalized gradient approximation made simple. Phys. Rev. Lett. **77**, 3865–3868 (1996).

[31] Gao, J., Wu, Q., Persson, C. & Wang, Z. Irvsp: to obtain irreducible representations of electronic states in the VASP. arXiv:2002.04032 (2020).

[32] Pezzini, S., Van Delft, M. R., Schoop, L. M., Lotsch, B. V., Carrington, A., Katsnelson, M. I., Hussey, N. E. & Wiedmann, S. Unconventional mass enhancement around the Dirac nodal loop in ZrSiS. Nat. Phys. **14**, 178–183 (2018).

[33] Shishido, H., Settai, R., Aoki, D., Ikeda, S., Nakawaki, H., Nakamura, N., Iizuka, T., Inada, Y., Sugiyama, K., Takeuchi, T., Kindo, K., Kobayashi, T. C., Haga, Y., Harima, H., Aoki, Y., Namiki, T., Sato, H. & Onuki, Y. Fermi surface, magnetic and superconducting properties of $LaRhIn_5$ and $CeTIn_5$ (T: Co, Rh and Ir). J. Phys. Soc. Jpn. **71**, 162–173 (2002).

[34] Koster, G.F., Dimmock, J.O., Wheeler, R.G. & Statz, H. Properties of the Thirty-Two Point Groups (1963), the point group citations: C_{2v} in Table 17 on page 36, C_{4v} in Table 33 on page 45 .

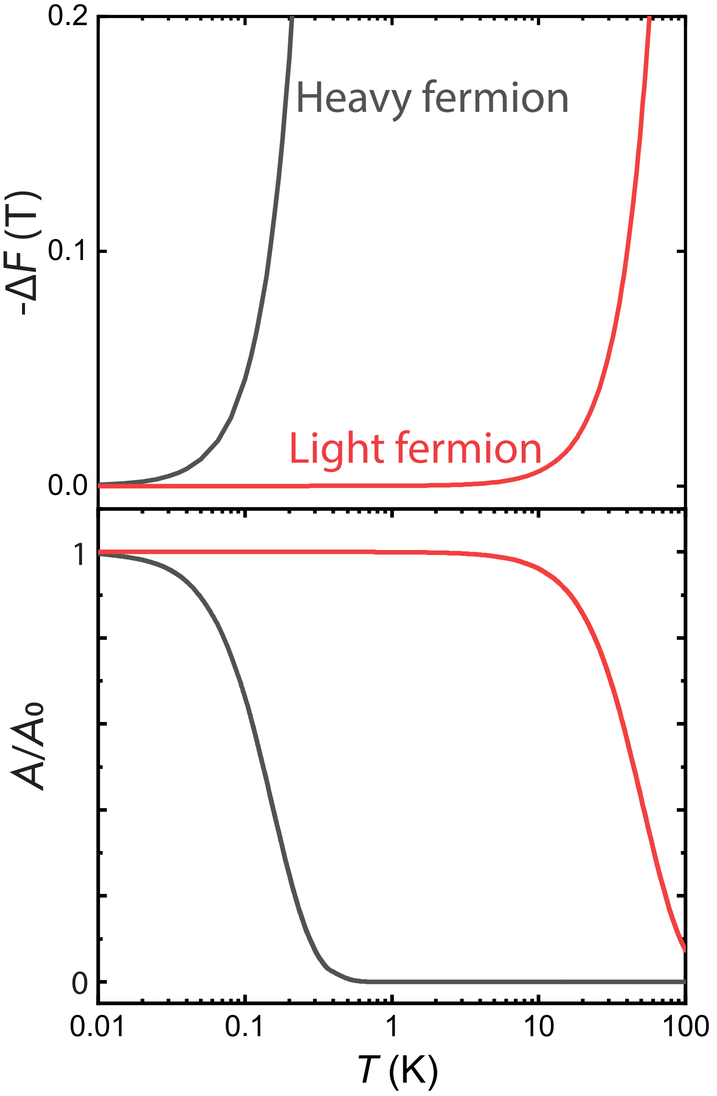

Supplementary Figure 1. Illustration of frequency change vesus smearing of oscillation amplitude due to increasing temperature. Here zero-temperature oscillation frequency (F_0) is chosen to be 50 T for both cases, while cyclotron mass is $10m_e$ and 0.03 m_e for heavy and light fermion respectively.

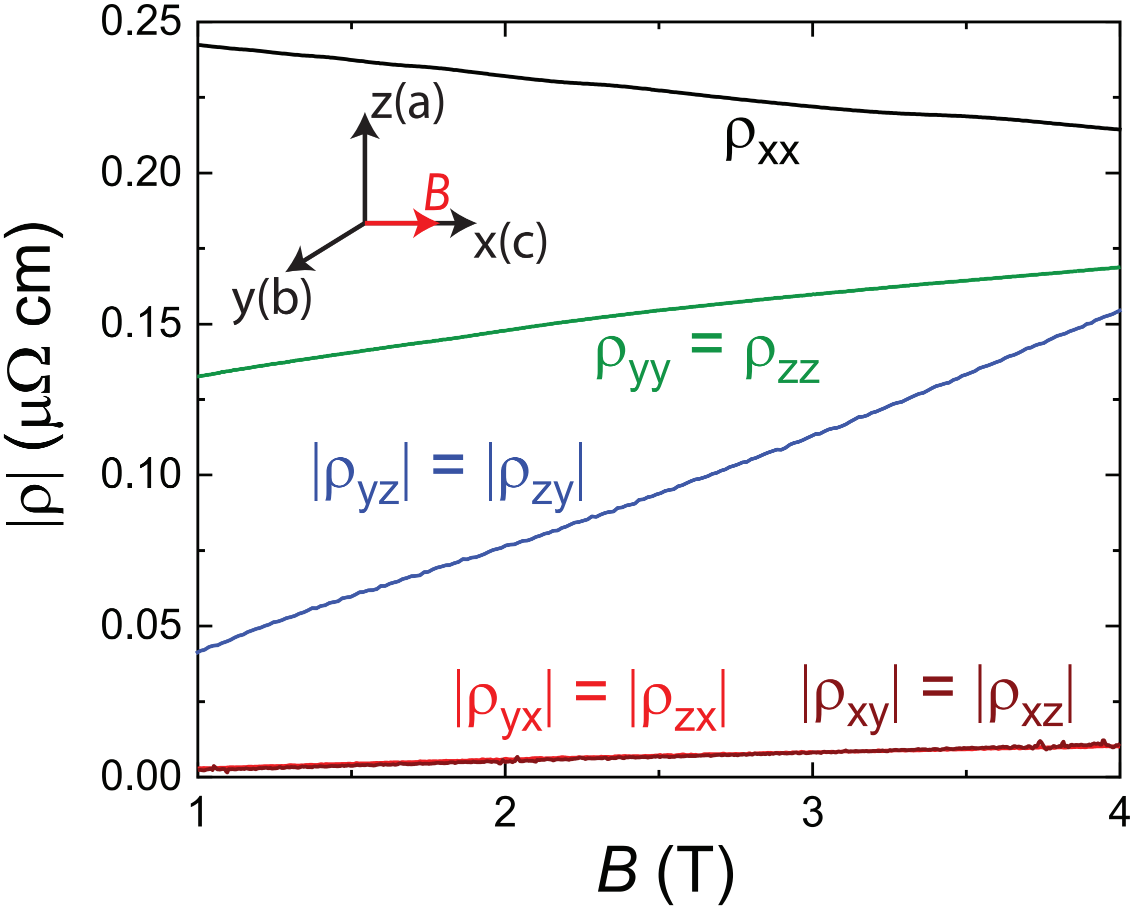

Supplementary Figure 2. All components of resistivity tensor of $LaRhIn_5$ measured with magnetic field applied along $x(c)$-axis. The planar Hall components $(\rho_{xy}, \rho_{xz}, \rho_{yx}, \rho_{zx})$ is much smaller than the Hall resistivity(ρ_{yz}, ρ_{zy}) and magnetoresistivity$(\rho_{xx}, \rho_{yy}, \rho_{zz})$ components.

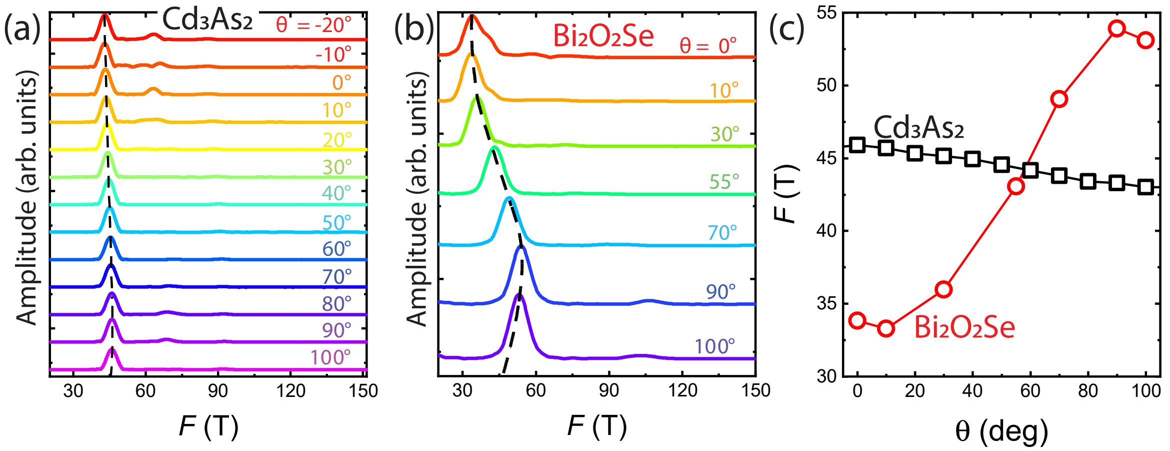

Supplementary Figure 3. The Fast-Fourier-Transformation (FFT) spectrum at different angles for Cd_3As_2 and Bi_2O_2Se respectively. (a) For Cd_3As_2, θ stands for the angle between applied field and [111]-axis. (b) For Bi_2O_2Se, $\theta = 0°$ is defined as field applied along c-axis.

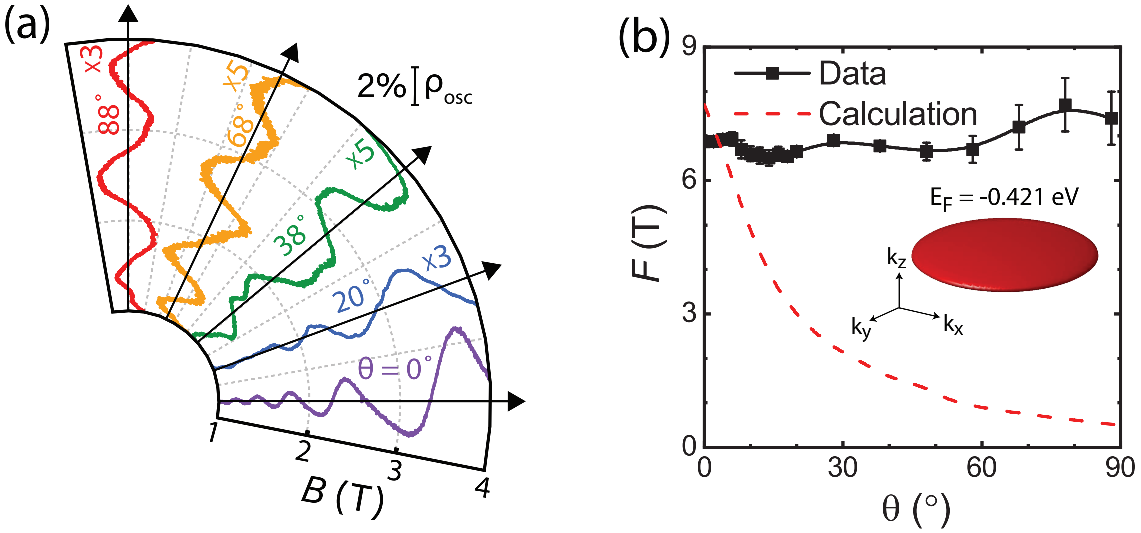

Supplementary Figure 4. Angle-dependent oscillation frequency corresponds to the small Dirac pocket in $LaRhIn_5$. (a) Fan plot of angle-dependent quantum oscillations ($F \approx 7$ T) at $T = 2$ K. θ is the angle between the magnetic field and the c-axis. (b) Angular dependence of measured oscillation frequency (black) deviates from the ab-initio calculation (red). The error bar is defined by the standard error of the fitting parameters generated by the fitting of quantum oscillations presented in (a). The calculated Fermi surface of the Dirac node at E_F = -0.421 eV is illustrated in the inset.

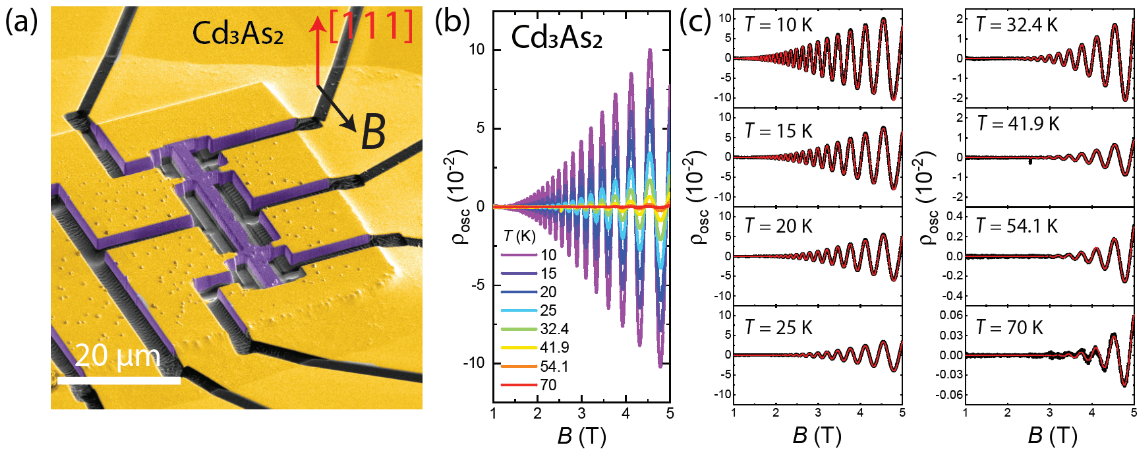

Supplementary Figure 5. Quantum oscillation measurements of Cd_3As_2. (a) Scanning Electron beam micrograph of a FIB prepared Cd_3As_2 microstructure. The crystalline pathway (purple) is electrically connected via gold top contacts (yellow). The magnetic field is applied perpendicular to the out-of-plane [111] axis. (b) temperature dependent quantum oscillations, (c) comparison of the experimental results (black curve) to the LK fit (red) at all temperatures.

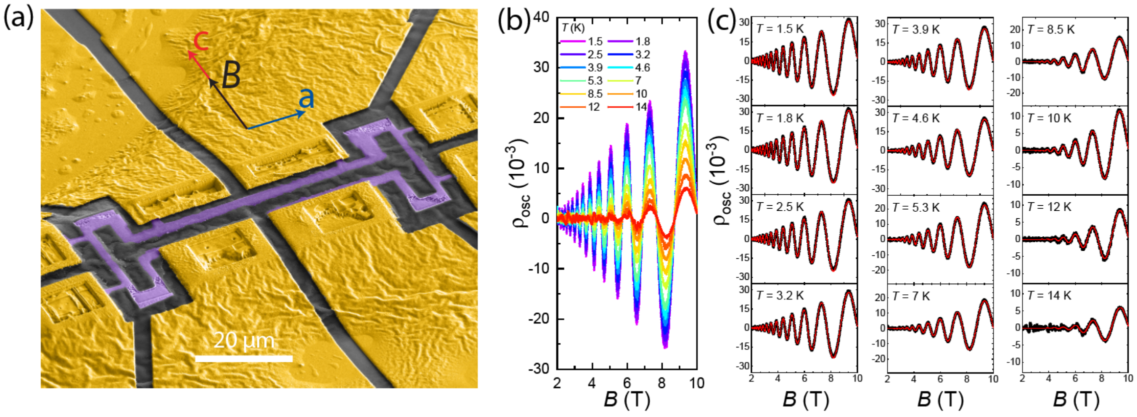

Supplementary Figure 6. Quantum oscillation measurements of Bi_2O_2Se. (a) Scanning Electron beam micrographs of the Bi_2O_2Se device. The magnetic field is applied along *c*-axis.(b) temperature dependent quantum oscillations, (c) comparison of the experimental results (black curve) to the LK fit (red) at all temperatures.

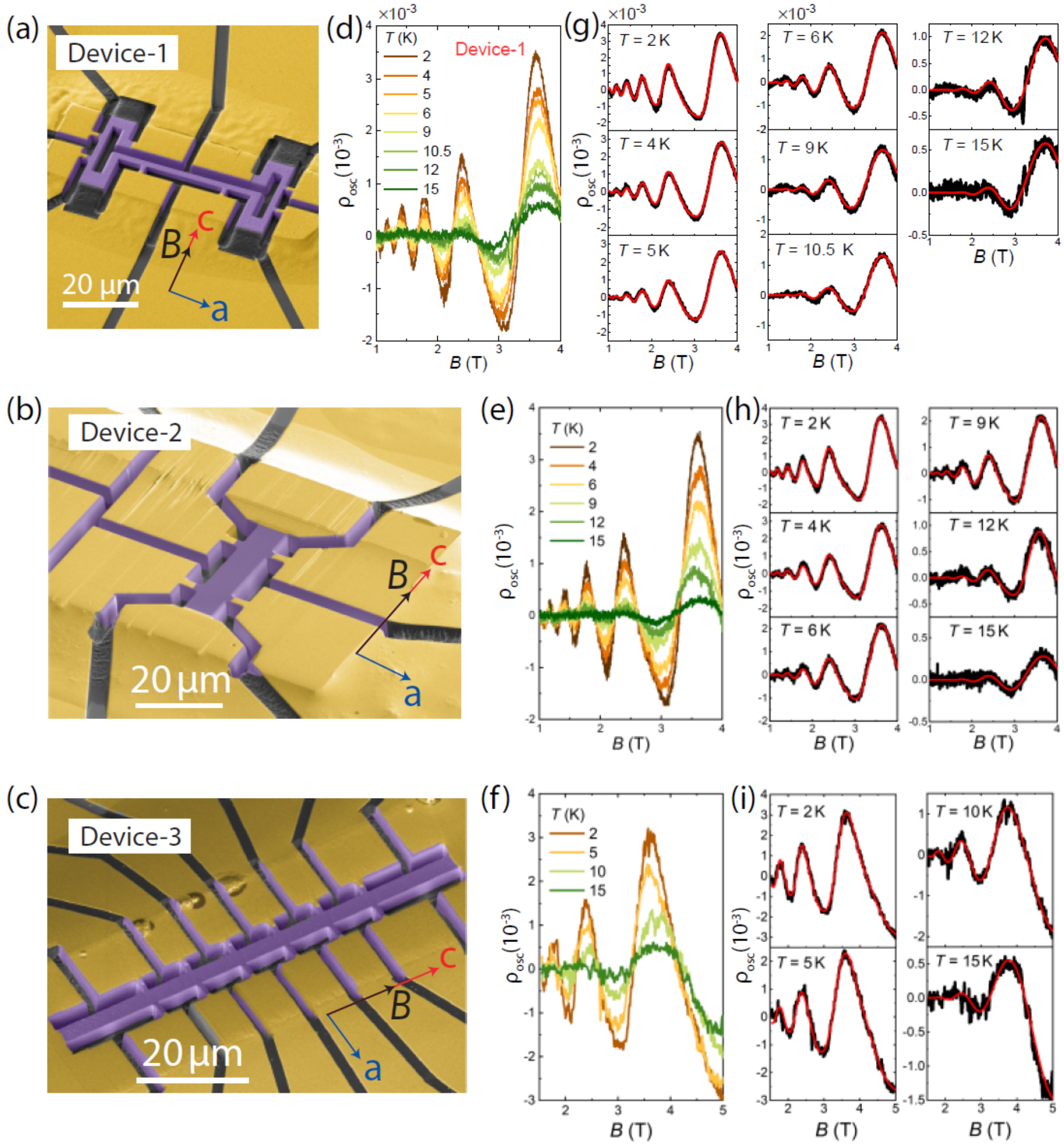

Supplementary Figure 7. Quantum oscillation measurements of $LaRhIn_5$. (a), (b) and (c) display the Scanning Electron beam micrographs of devices 1-3 of $LaRhIn_5$ respectively. (d), (e) and (f) present the temperature dependent quantum oscillations, while (g), (h) and (i) show the fits (red curve) to the experimental results (black) at all temperatures.

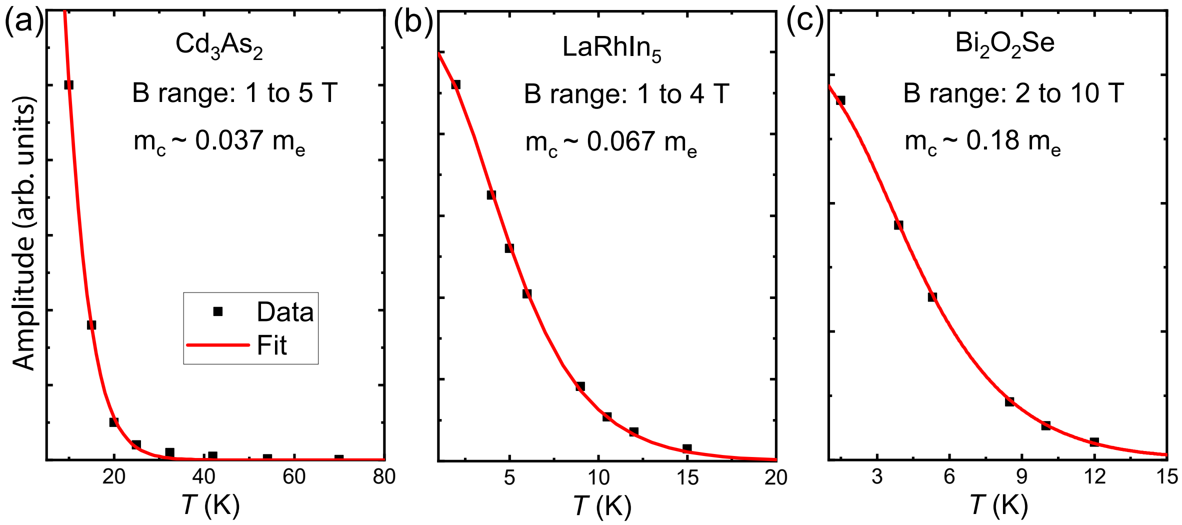

Supplementary Figure 8. Temperature dependence of oscillation amplitude for Cd_3As_2 (a), $LaRhIn_5$ (b) and Bi_2O_2Se (c). The fitting yields consistent value of cyclotron mass with the direct fitting described in Sec.

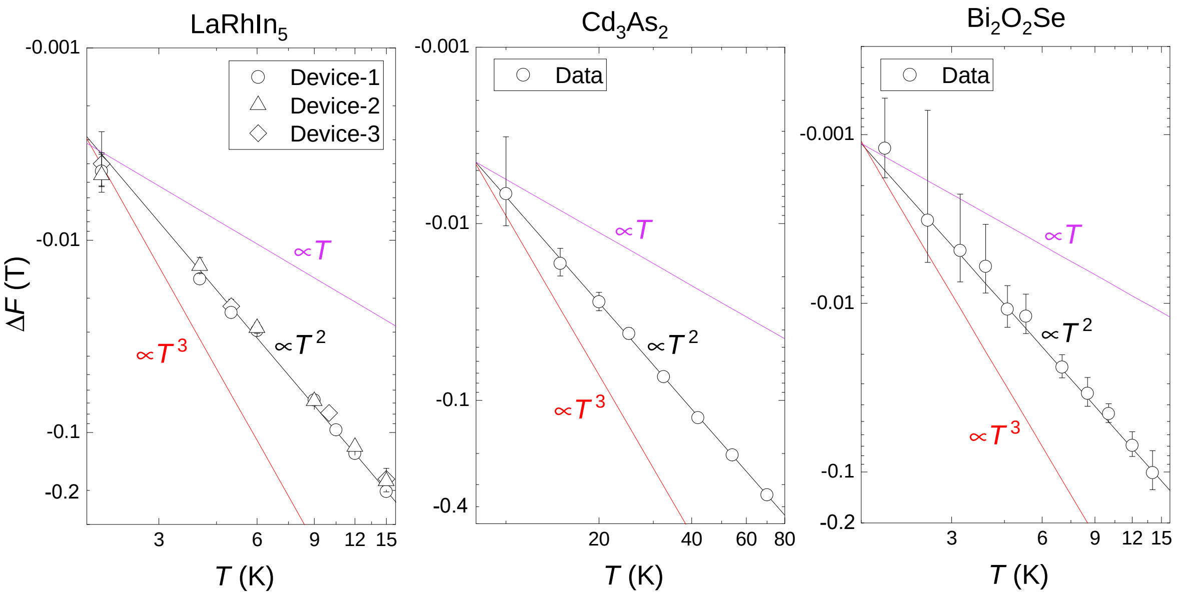

Supplementary Figure 9. Log-log plot of temperature dependence of oscillation frequency change ΔF for $LaRhIn_5$, Cd_3As_2 and Bi_2O_2Se. The error bar is determined by the standard error of the fitting parameters generated by the non-linear regression fitting procedure. It is clear that T^2 law is the only meaningful description of our data.

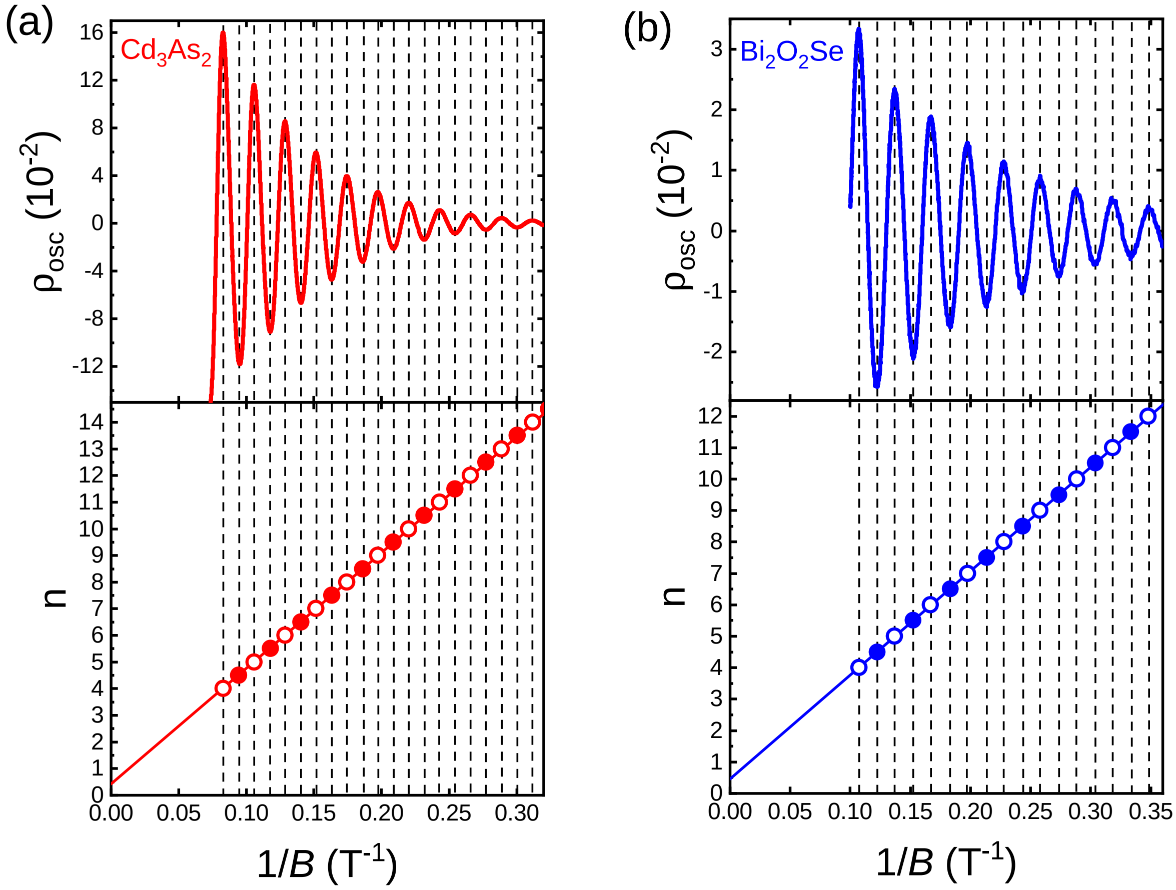

Supplementary Figure 10. Landau-fan plot of Cd_3As_2 (a) and Bi_2O_2Se (b) respectively. For SdH oscillations measured with the longitudinal configuration, the peaks of the oscillations correspond to the integer number(open symbol) of Landau index, while the valleys correspond to the half-integer (solid symbol) ones. The analysis for both materials demonstrates a residual Landau index close to 0.5, which may be incorrectly interpreted as both materials being "topological", while Bi_2O_2Se is clearly topologically-trivial.

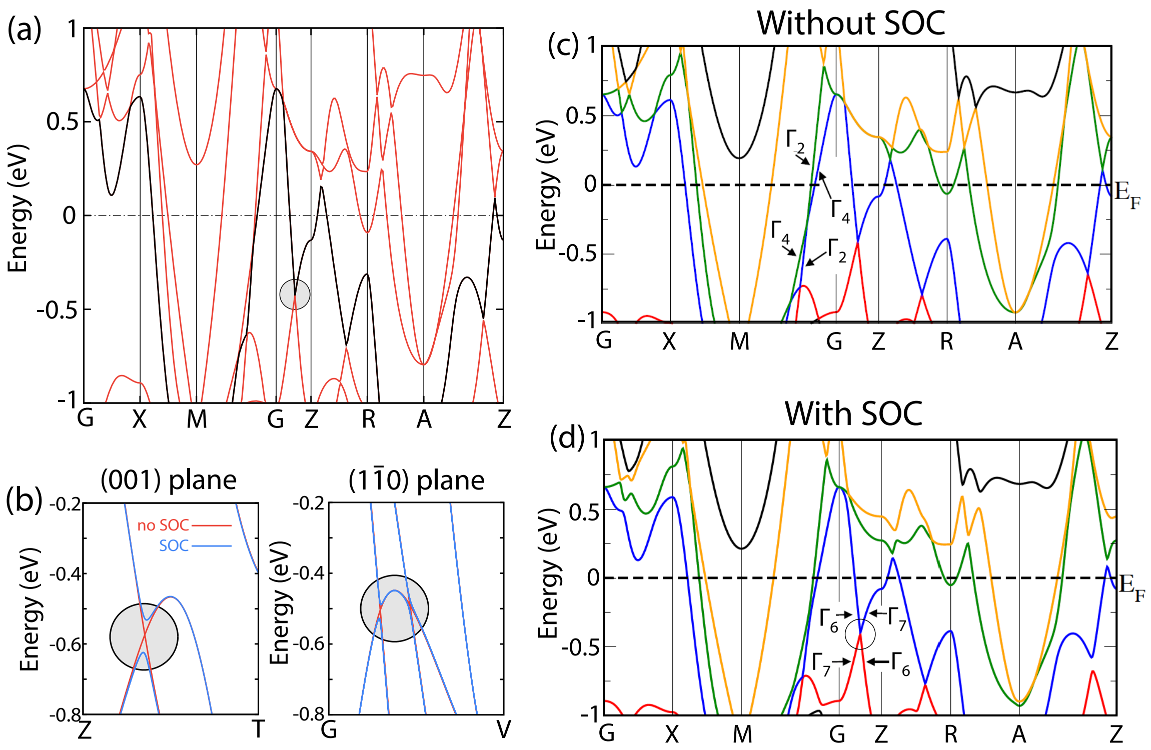

Supplementary Figure 11. Band structure calculations for $LaRhIn_5$. (a) Ab-initio-calculated band structure of $LaRhIn_5$, with a 3D Dirac point encircled. (b) illustrates the SOC-induced energy gaps at two cross-sections of the nodal line. (c) and (d) compares band structure of $LaRhIn_5$ with and without spin-orbit coupling calculated with the consideration of PBE. Γ_2 and Γ_4 in panel (c) are two irreducible representations of little point group C_{2v}. Γ_6 and Γ_7 in panel (d) are two irreducible representations of double point group C_{4v}[34].

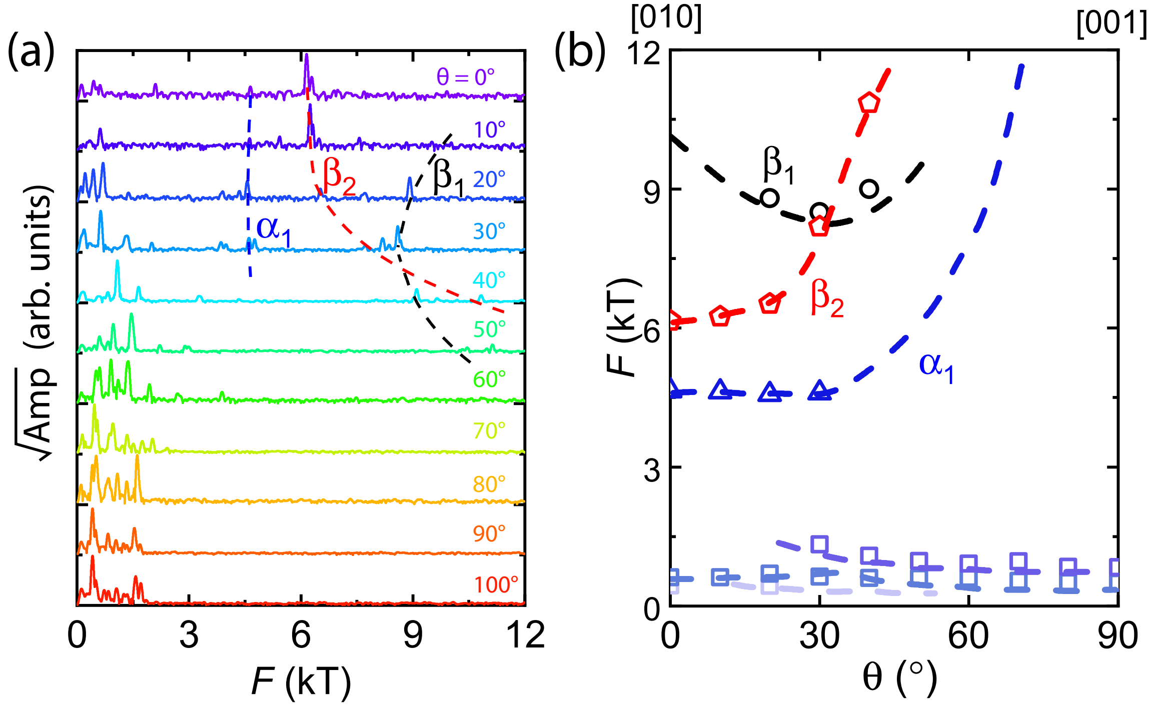

Supplementary Figure 12. Large-orbit quantum oscillations in $LaRhIn_5$. (a) Angle-dependent FFT spectrum for SdH oscillations with a field window of 8 to 14 T. The angle θ stands for the angle between magnetic field and c-axis. (b) Angular dependence of oscillation frequencies. Here θ is the angle between the applied magnetic field and the c axis. The solid symbols represent our results, while the results digitized from ref.[33] are displayed as the dashed lines. These results indicate that the high crystalline quality of the bulk crystals was not impacted by the microfabrication procedure.

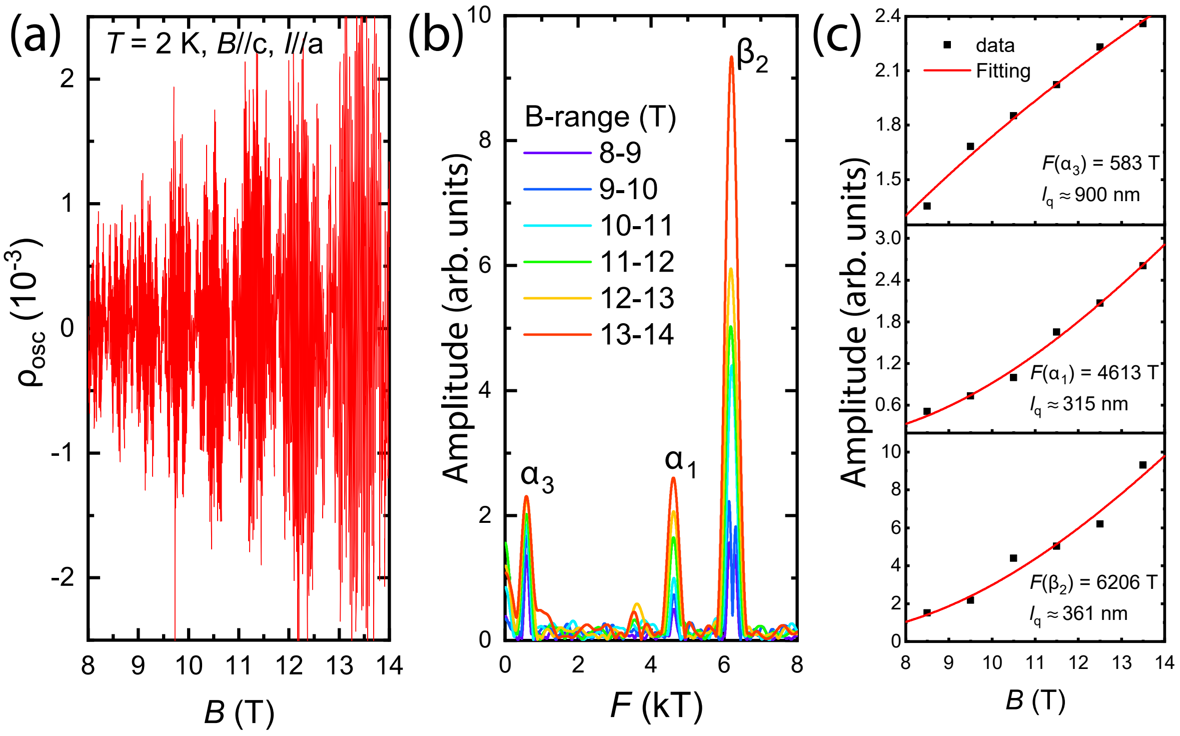

Supplementary Figure 13. Quantum mean free path analysis in $LaRhIn_5$. (a) Observation of clear high frequency SdH oscillations at $T = 2$ K with field and current applied along the c-axis. The observation of these high frequencies requires magnetic field in excess of the quantum limit of the small pocket, which hence is not observable here. (b) FFT spectrum at $T = 2$ K for different field windows. Three major peaks, which corresponds to α_3, α_1 and β_2 orbits are clearly observed. (c) The value of quantum mean free path (l_q) is obtained by Dingle analysis. The results suggest long quantum mean free paths.
